# Awareness of Medical Professionals Regarding Research Ethics in a Tertiary Care Hospital in Riyadh, Saudi Arabia: A Survey to Assess Training Needs

**DOI:** 10.3390/healthcare11202718

**Published:** 2023-10-12

**Authors:** Ghiath Alahmad, Khalid Malawi Alshahrani, Renad Abdulaziz Alduhaim, Rawan Alhelal, Rawa M. Faden, Naila A. Shaheen

**Affiliations:** 1 King Abdullah International Medical Research Center (KAIMRC), King Saud Bin Abdulaziz University for Health Sciences, Riyadh 11481, Saudi Arabia; alshahrani250@ksau-hs.edu.sa (K.M.A.); alhelal204@ksau-hs.edu.sa (R.A.); faden024@ksau-hs.edu.sa (R.M.F.); ashrafna@mngha.med.sa (N.A.S.); 2 College of Medicine, Imam Mohammad Ibn Saud Islamic University, Riyadh 11432, Saudi Arabia; 441020699@sm.imamu.edu.sa

**Keywords:** research, ethics, awareness, informed consent

## Abstract

Background: Ethics is an essential component of human research, and knowledge and awareness of ethical guidelines are required to conduct research involving human subjects and ensure the participants’ safety. Objectives: To investigate medical professionals’ and researchers’ knowledge of national and international research ethics guidelines, key principles in human research projects, ethical issues in different types of research, the importance of informed consent, and institutional review boards. Materials and methods: A cross-sectional study with 251 participants, including physicians, nurses, and researchers from three different research centers and hospitals in Riyadh city, was carried out using an electronic self-structured questionnaire. The sample size was estimated using OpenEpi, Version 3. The questionnaire contained six different sets of questions to analyze knowledge about research ethics and guidelines. The results were analyzed using SAS version 9.4 (SAS Institute Inc., Cary, NC, USA). Results: About 53.78% of the participants had received research ethics education, out of which 78.51% of the participants expressed the need for more training in research ethics education. The Belmont Report showed a high level of unawareness among nurses (88.3%), physicians (73.8%), and researchers (55.32%). Nurses had a high level of positive attitude about all the key principles of human research. The highest level of awareness of ethical issues in clinical trials and interventional studies was found among researchers (54%), and the lowest level of awareness was exhibited by nurses (19.32%). Nurses (74.34%) and physicians (62.79%) had the lowest positive attitudes about obtaining children’s assent in pediatric research. Physicians and researchers had a higher awareness of the IRB than nurses. Conclusion: Researchers were well-versed in research ethics, regulatory guidelines, and ethical issues in various types of research, whereas nurses were enthusiastic about key principles in human research and the importance of informed consent. This study shows that a very low percentage of nurses have received research ethics education and emphasizes the critical importance of including research ethics education in nursing curricula, as the involvement of nurses in research is inevitable. This study also shows the lack of knowledge among nurses, researchers, and doctors about various international guidelines. It emphasizes the importance of adding regulatory guidelines to the curriculum and teaching them effectively to students. Also, periodic workshops should be conducted to enhance the practical knowledge of the professionals regarding the guidelines and guide them in overcoming the practical difficulties they encounter during their practice.

## 1. Introduction

Ethics is an integral part of research, especially when involving human subjects. In the early 1900s, there were no laws regulating human research ethics, and the consequences of this have been many violations of human rights in research. For instance, in 1789, Edward Jenner performed untested and unsafe medical procedures on human subjects; the Tuskegee Syphilis Study is another example of unethical research conducted on African American men [1]. Following these ethical malpractices in human research, an ethical code of practice for research was developed. The Nuremberg Code, established in 1948, was the first international document that emphasized voluntary participation and informed consent. The World Medical Association issued guidance in 1964 to help physicians while conducting biomedical research on humans. The Declaration of Helsinki regulates worldwide research ethics and establishes standards for “non-therapeutic research” and “research combined with clinical treatment”. The Declaration of Helsinki serves as the foundation for current Good Clinical Practices (GCPs). Later, in 1979, the Belmont Report was published, emphasizing basic ethical principles and guidelines for conducting research with human subjects [2].

Apart from the aforementioned international standards, many other international and local laws and regulations, such as the International Ethical Guidelines for Biomedical Research Involving Human Subjects [3], the International Conference on Harmonization (ICH) guidelines for Good Clinical Practices (GCPs) [4], and others, began to emerge. In Saudi Arabia, as in other developed countries and countries in the Middle East, an increasing interest in conducting various types of research has been accompanied by an equal interest in developing ethical standards [5,6]. So the National Committee of Bioethics was established in 2001, and the Saudi Law of Research on Living Creatures was announced in 2010, followed by the first edition of implementing regulations in 2011 and the second edition in 2016 [7,8]. Also, the Saudi Food and Drug Authority announced the regulations and requirements for conducting clinical trials on drugs [9,10]. Despite the fact that research ethics are a crucial component, researchers’ understanding of research ethics and awareness of various guidelines are inadequate [11]. Awareness of research ethics should not be limited to regulations and guidelines but also include understanding the principles and their applications, especially informed consent [12], and the institutional review board (IRB) [5]. Moreover, different types of research, such as genetic [7,13], qualitative [14], quantitative [15], and clinical trials [16], may have different ethical challenges. The ethical declarations and guidelines offer comprehensive information about the legal aspects of research and the appropriate conduct to be followed while conducting studies with human participants. Therefore, it is crucial for researchers or professionals involved in research to possess a comprehensive understanding of these standards.

With the expansion and rising interest in research ethics in Saudi Arabia, many institutes have become interested in providing training in research ethics. For example, King Abdullah International Medical Research Center (KAIMRC) has various activities, such as the Research Ethics Course, Research Coordinator programs, and the Research Summer School (RSS), which provides classes on medical research ethics [17]. Also, the ethics committee at KAIMRC supervises the research in all three institutes, KAIMRC, King Abdulaziz Medical City (KAMC), and King Abdullah Specialized Children Hospital (KASCH), that were included in our study. Other institutes provide similar activities as well, such as the King Faisal Specialist Hospital and Research Center (KFSHRC) in Riyadh [18]. Assessing the awareness and need for training in research ethics is essential to developing and improving good training programs.

The practice of ethical behavior is essential within the domain of medical research, which encompasses the involvement of human subjects. Numerous institutions worldwide have established dedicated committees to address and endorse activities with ethical implications, overseeing research conduct through national and/or regional review committees. On the other hand, countries with lower levels of development around the globe are making efforts to establish committees tasked with governing their academic and scientific endeavors. Research ethics committees’ main goals are to evaluate study proposals, try to understand why human or animal subjects are being used, figure out the pros and cons of using human or animal subjects (based on the balance between risks and possible benefits), and make sure that local ethical rules are followed. Therefore, all individuals engaged in the research project must possess a comprehensive understanding of the regulatory rules and principles governing their work to ensure the research’s ethical conduct [19].

Searching the literature using PubMed did not reveal any studies carried out to assess the awareness of medical professionals and researchers regarding research ethics in Saudi Arabia. However, few studies have been conducted in other Arab countries and other places. This is the first cross-sectional survey conducted in Saudi Arabia to assess awareness about research ethics among medical professionals and researchers. Therefore, in this cross-sectional observational study, we investigated and explored knowledge and awareness about research ethics and the level of satisfaction with ethics education, the need for training, and knowledge about regulatory guidelines. We also conducted extensive research about the ethical challenges faced in different kinds of research and awareness about informed consent and the IRB among medical professionals working in hospitals in Riyadh.

Aim of the study: This study aims to explore and assess the research ethics training needs among medical professionals in the King Abdullah International Medical Research Center (KAIMRC), King Abdulaziz Medical City (KAMC), and King Abdullah Specialized Children Hospital (KASCH) in Riyadh, Saudi Arabia.

### Specific Objectives

- To assess research ethics awareness among medical professionals and researchers.
- To explore and assess the awareness and training needs pertaining to international and national regulations on research ethics, the fundamental principles of medical research ethics, ethical issues, and practical implementations of research ethics across various research methodologies (genetic, qualitative, quantitative, and clinical trials), the significance of informed consent, and Institutional Review Board (IRB) oversight.
- To compare the research ethics awareness and training needs among different professional groups, including physicians, nurses, and researchers.

## 2. Materials and Methods

### 2.1. Study Design 

This is an observational, cross-sectional study. The electronic form of a self-structured questionnaire was distributed using tablets and was designed to assess professionals’ awareness of medical research ethics. 

### 2.2. Setting

The study was conducted among healthcare professionals and researchers at King Abdullah International Medical Research Center (KAIMRC), King Abdulaziz Medical City (KAMC), and King Abdullah Specialized Children Hospital (KASCH) in Riyadh, Saudi Arabia, from 4 August until 11 August 2022. The questionnaire was prepared in English. On average, filling out the questionnaire required 20 min. The entire process of questionnaire administration and collection of filled-out questionnaires took approximately one week.

### 2.3. Participants

**Figure 1:**
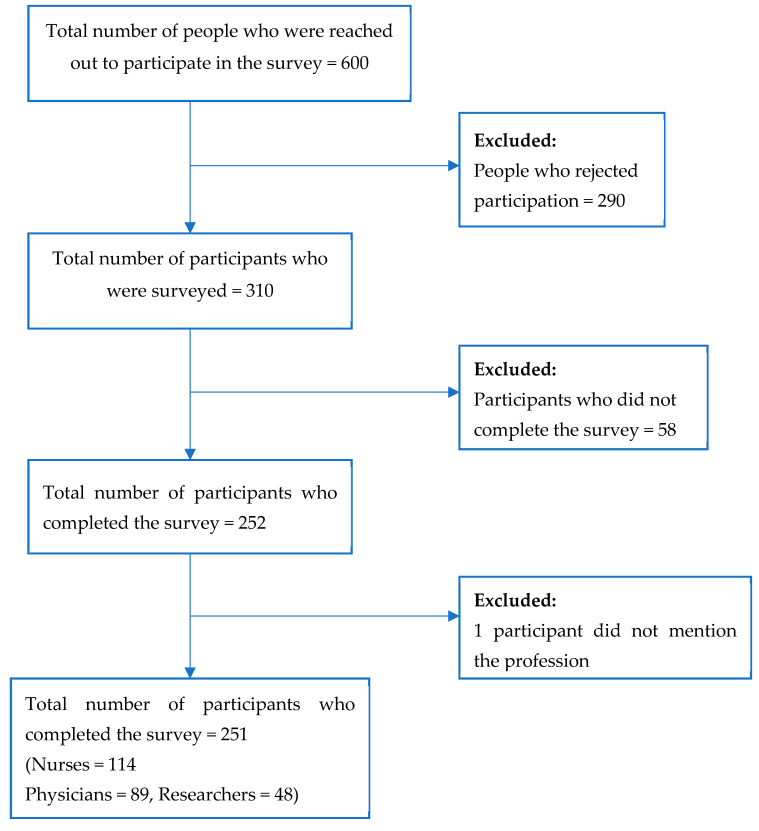
Schematic representation of the participant selection.

The whole sample size consisted of 251 individuals, encompassing medical practitioners, nursing professionals, and researchers. The nurses and physicians are affiliated with various healthcare divisions. These groups were chosen since they were commonly involved in research, and assessing their awareness would help guide the requirements of ethical education in research. Professionals who are not interested in medical research were not included in the study. The schematic representation of the participant selection is represented in Figure 1.

### 2.4. Variables

The questionnaire includes six domains:

- Socio-demographic data: The initial section of the questionnaire was designed to gather socio-demographic data from the participants, which included personal information about the participants’ social status, demographic status, educational, and research background.
- Regulations: This section was intended to gather data about the level of awareness of regulatory texts. The survey in this study utilized a Likert-type scale consisting of three response options.
- Principles: This section sought to gather data on the level of awareness pertaining to different research principles. The survey utilized a 4-point Likert-type scale, where respondents were asked to rate the importance of the items on a scale ranging from 1 (indicating low importance) to 4 (indicating high importance).
- Ethical challenges: This 3-point Likert scale survey was aimed at collecting information about their level of awareness about ethical challenges in several types of research designs.
- Informed consent: In this section, participants were requested to ascertain whether some aspects of informed consent are always, sometimes, or never true.
- IRB: This is a 5-point Likert-type survey, where respondents indicate their level of agreement on a scale ranging from 1 (strongly disagree) to 5 (strongly agree). In this section, participants were asked to determine whether they strongly agree, agree, are neutral, disagree, or strongly disagree with the listed information about the IRB.

### 2.5. Data Sources and Measurements 

The raw data were processed in accordance with the best practices for raw data management to identify any inaccuracies or incompleteness in advance of the statistical analysis. In order to fulfill this task, all interval variables were checked and summarized in terms of maximum and minimum values. Maximum and minimum values were checked and compared against the nominal maximum and minimum values of each variable, and variables with implausible values were flagged. A similar process was applied to categorical variables to identify any potential anomalies. All identified anomalies were corrected prior to the statistical analysis in accordance with the best practices for raw data management to identify any inaccuracies or incompleteness in advance of the analysis.

### 2.6. Bias

All eligible participants were independent, and they voluntarily agreed to participate with no external pressure. They were asked to complete the questionnaire immediately after signing the consent form. The validity of the questionnaires was assessed by conducting a pilot study with a sample of 10 participants to ascertain the clarity of the content. The IRB at King Abdullah International Medical Research Center (KAIMRC) approved the questionnaire form beforehand as acceptable.

### 2.7. Study Size

Based on healthcare professionals and researchers who have had 50% awareness of medical research ethics, assuming a finite population of 600, confidence limits of 95%, and precision of 5%, we anticipated that this study would require 235 participants. The sample size was estimated using OpenEpi, Version 2.3.1 [20]. Equation used to calculate sample size n = [DEFF × Np(1 − p)]/[(d2/Z21 − α/2 × (N − 1) + p × (1 − p)].

### 2.8. Statistical Methods

The results were analyzed using SAS version 9.4 (SAS Institute Inc., Cary, NC, USA) [21]. The categorical variables were summarized as frequencies and percentages, whereas continuous variables such as age were summarized as means and standard deviations, Chi-square and Fisher exact tests were used for comparison across groups (physicians, nurses, and researchers). The Wilcoxon rank-sum test was used to compare across groups. Significance was declared using an alpha less than 0.05. Moreover, the analysis was reported while disregarding the missing responses to some questions by several subjects. The research ethics awareness was reported as a frequency and percentage, as well as compared across groups by using the chi-square test. A *p*-value less than 0.05 was considered significant. The training needs of medical professionals were summarized as frequencies and percentages. To compare the training needs among different professional groups, including physicians, nurses, and researchers, the chi-square test was used, and a *p*-value less than 0.05 was considered significant. Logistic regression analysis was used to identify the predictors of awareness among the professionals. The list of independent variables included age, gender, profession, and training. Results were reported as OR, 95% CI, and *p*-value.

## 3. Results

### 3.1. Participants

A total of 600 participants were asked to take part in the survey, of whom 290 rejected participation. A total of 310 respondents filled out the survey. Fifty-eight participants did not complete the questionnaire, and one participant did not mention their profession. Finally, 251 questionnaires were included in the analysis, including 114 nurses, 89 physicians, and 48 researchers. Fifty-eight participants were excluded due to rejection or not completing the survey. The mean age of the participants was 34.25 ± 9.07. Most of the participants were female (64.54%), and most of them were nurses (45%). Most of the nurses (91.89%) and doctors (63.22%) had a bachelor’s level of education, whereas most of the researchers’ level of education was PhD (56.25%). 

### 3.2. Demographic Characteristics

About 53.78% of participants had previous training in research ethics, of whom 33.33% had it during their academic milieu, and 32.59% of the participants had received more than one type of training in research ethics. Compared to physicians and nurses, a higher percentage of researchers had received training in research ethics (79.17%), and about 60.5% of researchers had received more than one form of training in research ethics. Among physicians who have received training in research ethics, a higher percentage have received training through their academic curriculum (41.27%). Among the participants who had received training, the level of satisfaction (very satisfied and moderately satisfied) was high among researchers (47.4%), followed by physicians (41.9%) and nurses (40.3%). About 78.5% of participants expressed the need for more training, and among respondents who had not received prior training, approximately 72.41% of participants expressed an interest in getting trained in research ethics. About 31.87% of participants have taken on responsibilities as principal investigators (PI) or co-principal investigators (Co-PI)**.** The demographic data are represented in Table 1.

### 3.3. Awareness of Key International and Local Regulatory Texts Guiding the Protection of Human Research Participants

**Figure 2:**
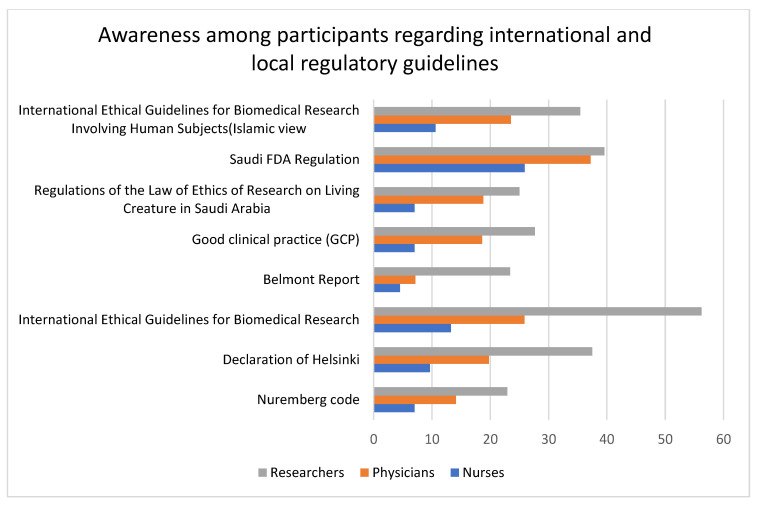
The bar graph represented here compares the level of awareness about various international and local regulatory guidelines among researchers, physicians, and nurses. The graph shows that researchers have a high level of awareness, while nurses have a low level of awareness, about all the guidelines. The highest level of awareness was seen for the international ethical guidelines for biomedical research among researchers. The Nuremberg code and Belmont Report have the lowest level of awareness.

**Figure 3:**
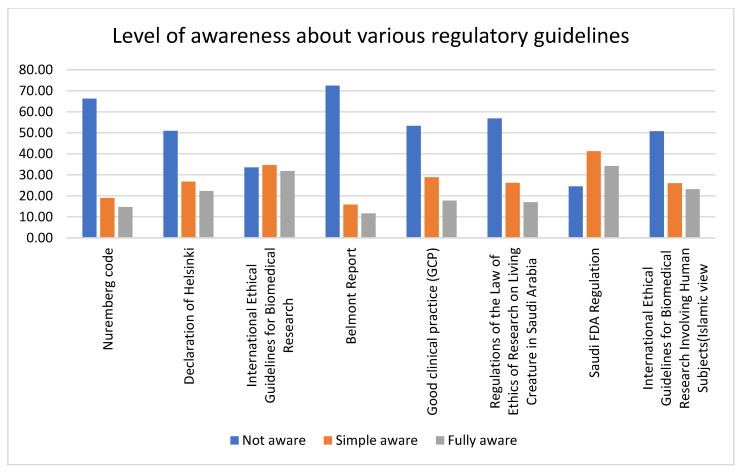
The bar graph represents the level of awareness among participants about various regulatory guidelines. Except for the Saudi FDA Regulation, all the other regulatory guidelines have a high level of unawareness, with the Belmont Report being the highest.

Nurses were mostly unaware, and researchers had high awareness, of almost all the regulatory texts. There was a high level of unawareness of the Belmont Report among nurses (88.3%), physicians (73.8%), and researchers (55.32%), followed by the Nuremberg Code (nurses = 84.2%, physicians = 64.7%, and researchers = 50%) and regulations of the law of ethics of research on living creatures in Saudi Arabia (nurses = 78.9%, physicians = 54.1%, and researchers = 37.5%). Among researchers, the level of awareness about international ethical guidelines for biomedical research (56.3%) was high. The nurses had a better level of awareness about the Saudi FDA Regulation (25.9%) compared to other guidelines. Also, the level of awareness was lower for only the Saudi FDA regulation, whereas all the other guidelines had a higher level of unawareness. In general, nurses had the lowest level of awareness, and researchers had the highest level of awareness, about both the national and international regulatory texts. All the variables were statistically significant. Data about the level of awareness about various regulatory guidelines across professions are given in Table 2 and represented in Figure 2 and Figure 3.

### 3.4. Attitude toward the Existence, Significance, and Capacity to Implement Key Principles Guiding the Protection of Human Research Subjects

**Figure 4:**
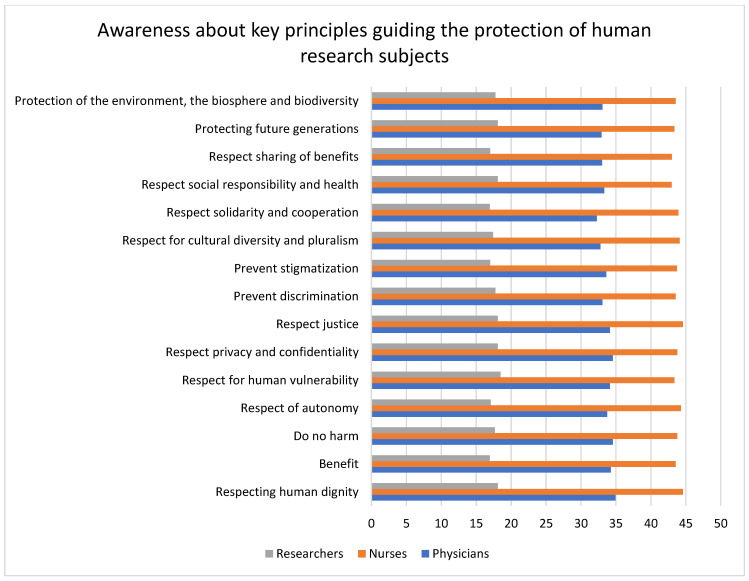
The bar graph illustrated here compares the attitudes toward the key principles guiding the protection of human research subjects among researchers, physicians, and nurses. The graph shows that nurses had a high positive attitude, whereas researchers had the least positive attitude toward all the key principles, albeit the overall response being very low (not exceeding 50%).

Nurses had a high level of positive attitude toward all the key principles, followed by physicians, while researchers had the least positive attitude about all the key principles. Physicians had the most positive attitudes toward human dignity (34.9%), while nurses had the most positive attitudes toward solidarity and cooperation (44.9%). Researchers had a high positive attitude about respect for human vulnerability (18.4%). The principles of respect for autonomy (*p* = 0.03) and respect for solidarity and cooperation (*p* = 0.03) were statistically significant. The data are given in Table 3 and represented in Figure 4.

### 3.5. Awareness among Respondents of the Existence of Ethical Issues in Several Types of Research

**Figure 5:**
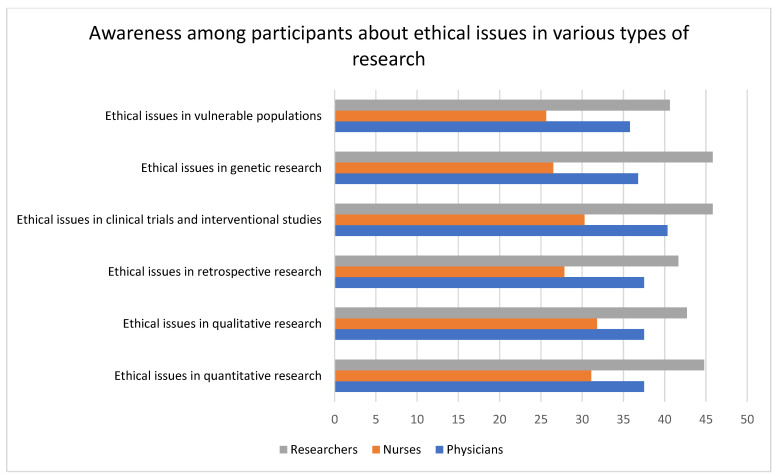
The bar graph illustrated here compares the awareness of ethical issues in various types of research among researchers, physicians, and nurses. Comparatively, the researchers had a high level of awareness, and nurses had a low level of awareness, for all the types of research, although the overall response was very low (not exceeding 50%).

**Figure 6:**
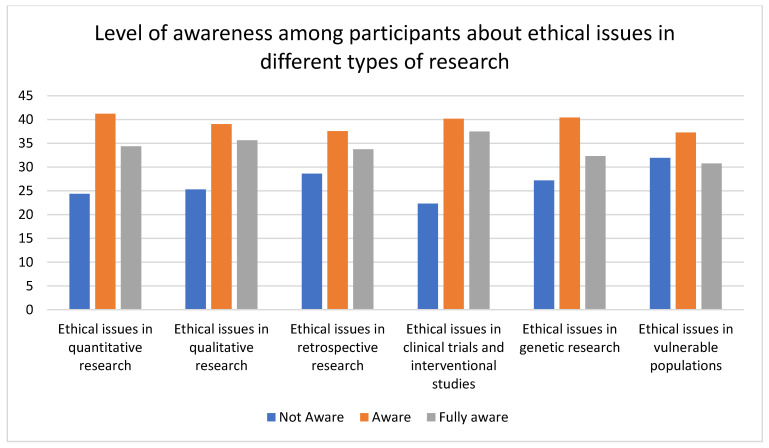
The bar graph represents the level of awareness among participants about ethical issues in different types of research. The graph shows that, on average, only about 40% of the participants were aware of the ethical issues in different types of research.

Researchers had the highest level of awareness, and nurses were unaware, of the ethical issues involved in various kinds of research. In total, the level of unawareness about ethical issues in research involving vulnerable populations was high (physicians = 28.41%, nurses = 48.67%, and researchers = 18.75%). Researchers showed the highest level of awareness of ethical issues in clinical trials and interventional studies (54%), and nurses showed the lowest level of awareness (19.32%). All the variables were statistically significant. The data are represented in Table 4 and Figure 5 and Figure 6.

### 3.6. Knowledge of Physicians, Nurses, and Researchers Regarding Informed Consent and Its Usage

**Figure 7:**
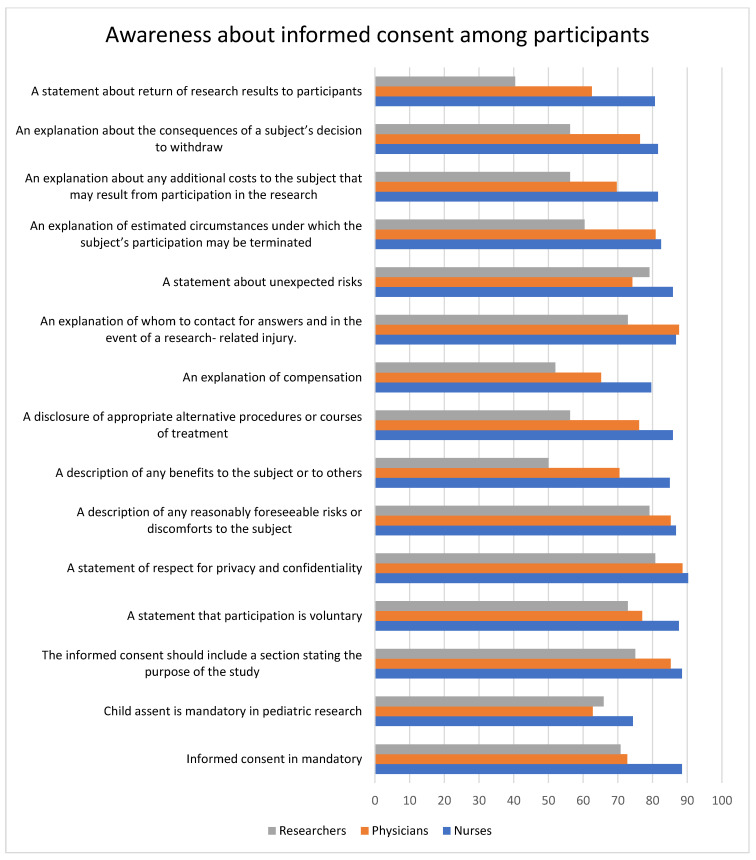
The bar graph illustrated here compares the attitudes toward informed consent among researchers, physicians, and nurses. The graph shows that nurses and physicians had a higher positive attitude toward informed consent compared to researchers.

**Figure 8:**
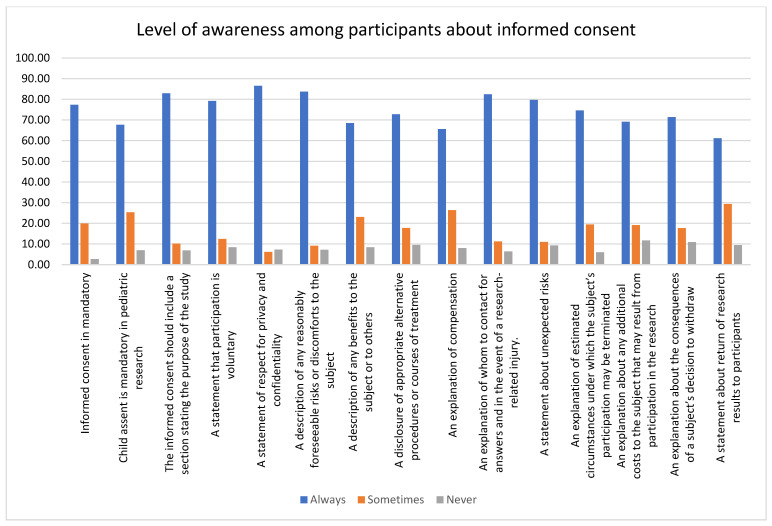
The bar graph represents the attitude toward informed consent among participants. On average, about 80% of the participants showed a positive attitude toward informed consent. The highest positive attitude was seen for respect for privacy and confidentiality, while the lowest positive attitude was seen for the return of research results.

The nurses had a positive attitude toward all the statements about the importance of informed consent except for the explanation of whom to contact for answers and, in the event of a research-related injury, only for which the physician had a high positive attitude. Researchers had the least knowledge about the importance of informed consent. The respect for privacy and confidentiality had the highest level of positive attitude among all the participants (nurses = 90.27%, physicians = 88.64%, and researchers = 80.8%). Nurses (74.34%) and physicians (62.79%) had the lowest positive attitude about obtaining children’s assent in pediatric research, whereas researchers had the least positive attitude about returning research results to participants (40.4%). Informed consent is mandatory (*p* = 0.001); the informed consent should include a section stating the purpose of the study (*p* = 0.05); a statement that participation is voluntary (*p* = 0.04); a description of any benefits to the subject or others (*p* < 0.0001); a disclosure of appropriate alternative procedures or courses of treatment (*p* = 0.0006); an explanation of compensation (*p* = 0.002); an explanation of estimated circumstances under which the subject’s participation may be terminated (*p* = 0.02); an explanation about the consequences of a subject’s decision to withdraw (*p* = 0.01); and a statement about the return of research results to participants (*p* < 0.0001) were all statistically significant where nurses had a high level of acceptance. The data are represented in Table 5 and Figure 7 and Figure 8.

### 3.7. Respondents’ Awareness of the Existence and Significance of the IRB Statements

**Figure 9:**
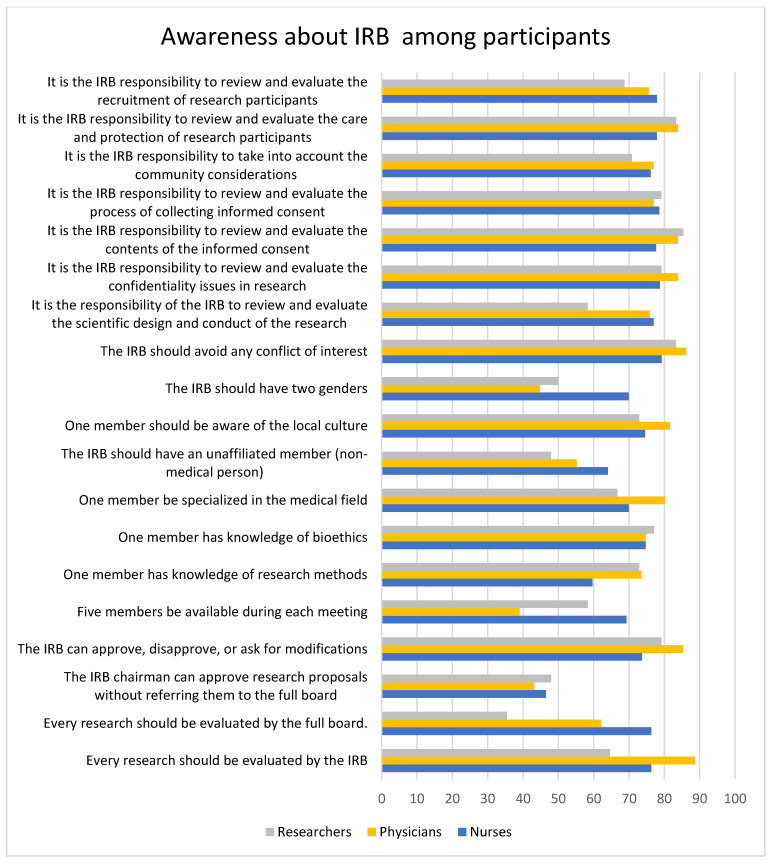
The bar graph represents the attitude toward IRB researchers, physicians, and nurses. Almost all the participants had a high positive attitude toward the IRB, except for the statement that the IRB chairman can approve research proposals without referring them to the full board, which had the least positive attitude among all the participants.

For most of the statements, the positive attitude was high among all the participants. Comparatively, physicians and researchers had a higher positive attitude toward most of the statements than nurses, except for the statement that the IRB should have two genders, which 69.9% of the nurses agreed to, and 44.8% and 50% of the physicians and researchers agreed to, respectively. The lowest percentage of positive attitude was seen for the statement that the IRB chairman can approve research proposals without referring them to the full board, which only 46.5%, 43.18%, and 47.92% of the nurses, physicians, and researchers agreed to, respectively. A total of 88.6% of physicians agreed that the IRB should review every research project, which was the most popular opinion among physicians. About 83.3% of the researchers strongly agreed with the statement that it is the IRB’s responsibility to review and evaluate the care and protection of research participants. The statements “every research should be evaluated by the IRB” (*p* = 0.0001), “every research should be evaluated by the full board” (*p* = 0.0001), “five members should be available during each meeting” (*p* = 0.0004), “the IRB should have two genders” (*p* = 0.0014), and “It is the responsibility of the IRB to review and evaluate the scientific design and conduct of the research” (*p* = 0.007) were all statistically significant, and nurses had a high level of acceptance of these statements, whereas physicians had high acceptance of the statistically significant statements “one member should be specialized in the medical field” (*p* = 0.04) and the IRB should avoid any conflict of interest” (*p* = 0.0005). “The IRB chairman can approve research proposals without referring them to the full board” (*p* = 0.02) and “It is the IRB responsibility to review and evaluate the process of collecting informed consent” (*p* = 0.04) were highly accepted by researchers. The data are represented in Table 6 and Figure 9.

## 4. Discussion

In this study, we investigated the education on research ethics, awareness of national and international guidelines on research ethics, key principles of research ethics, ethical issues in different types of research, attitude toward informed consent, and IRB among healthcare professionals and researchers at three different hospitals. 

### 4.1. Training and Education on Research Ethics among Participants

Among the participants, a higher percentage of researchers had received training in research ethics (79%). Our results reveal that compared to nurses and researchers, the majority of the physicians (41.27%) had received research ethics education through their academic curriculum. Despite the fact that nurses play an important role in clinical research, only a very low percentage of the nurses had training in research ethics. A survey by Aksoy et al. (2018) [22] reported that only 3.8% (*n* = 291) of the nurses had relevant training in research ethics, and 85.2% of the participants considered that clinical research should be included as a part of nursing education. This is consistent with our finding that about 80.6% of the nurses expressed that they wanted to receive training in research ethics. Researchers expressed a high level of satisfaction (very satisfied and moderately satisfied) about the training (47.37%). Though 43.33% of the participants were satisfied (very satisfied and moderately satisfied) with their training, 78.51% of the participants expressed the need for more training in research ethics. This is in line with the survey by Alotaibi et al. (2021) [23] among dental researchers, which found that many participants, especially general practitioners and consultants, lacked knowledge about research ethics and were in need of attending effective workshops. It was reported that students who received education outside Saudi Arabia have an increased awareness of research ethics and scientific approaches, and this underlines the necessity of implementing ethics education in university curricula [24]. A cross-sectional study of physicians’ attitudes and knowledge of research ethics in Lebanon discovered that only 27.4% were aware of the Lebanese National Consultative Committee on Ethics (LNCCE), and participants with a Ph.D. had a higher level of research ethics knowledge, which is consistent with our findings [25].

### 4.2. Awareness of Key International and Local Regulatory Guidelines

We next investigated the level of awareness about various international and local regulatory guidelines regarding the protection of human research participants. Nurses exhibited poor awareness of all the regulatory guidelines; comparatively, researchers exhibited a good level of awareness, but still, less than half of the researchers were aware of all the guidelines except for the International Ethical Guidelines for Biomedical Research. A study conducted in Japan by Yanagawa et al. (2014) [26] reported that less than 50% of the nurses were aware of the Declaration of Helsinki, ethical guidelines, Good Clinical Practice, institutional review boards, and ethics committees. A nurse’s role in clinical research cannot be eliminated. The promotion of clinical trials, the recruiting of patients for clinical trial participation, the education of the patient and family, and the clinical care and support of patients during their involvement in clinical trials are all crucial roles that nurses play [27]. However, our results show that nurses’ level of knowledge about the regulatory guidelines is inadequate. All the participants were highly unaware of the Nuremberg Code (66%), the Declaration of Helsinki (50.9%), and the Belmont Report (72.67%). In a survey by Alardan et al. (2021) [28] among family medicine trainees, it was reported that about 88.5% were unaware of the Nuremberg Code and 93.8% were unaware of the Helsinki Declaration. In another study reported by Al-Tannir et al. (2018) [29], about 56% of the respondents (clinicians conducting clinical trials) were completely unaware of the Declaration of Helsinki. On average, the participants had a better level of awareness about Saudi FDA Regulation (34.2%) and International Ethical Guidelines for Biomedical Research (31.8%) compared to other guidelines. In a survey by Almazrou et al. (2019) [11], only 4.3% (*n* = 254) of the participants were aware of the usage of the Saudi FDA as a drug information resource. In a study conducted in Jordan among clinicians, a majority of the participants had no knowledge about the Helsinki Declaration or the Belmont Report [30], which is consistent with our survey results.

### 4.3. Attitude toward the Key Principles Guiding the Protection of Human Research Subjects

The level of awareness regarding the key principles was next investigated. Adhering to the key principles is important to avoid any misconduct in research. All the participants had a low positive attitude toward the key principles of research ethics, with none of the responses exceeding 50%. Comparatively, nurses had a higher positive attitude toward all the key principles, whereas researchers had the least positive responses toward all the key ethical principles, with none of the responses exceeding 20%. In spite of the fact that researchers showed a high level of awareness toward the regulatory guidelines, they had the least positive attitude toward the key principles guiding human research subject protection. Researchers had a low positive attitude toward preventing discrimination. Few other studies have also reported the researchers’ bias in participant selection. Potential minority participants were not regarded as ideal study candidates because interactions with them were deemed difficult, denying them clinical trial opportunities and thereby discriminating against them [31]. A low positive attitude among participants was also seen for the principles of protecting future generations and protecting the environment, the biosphere, and biodiversity. The results are similar to the survey by Ahmad (2021) [32], where the healthcare professionals’ (HCPs’) knowledge about biomedical waste management was inadequate, and nurses, comparatively, had a higher awareness of biomedical waste management. 

### 4.4. Awareness of Ethical Issues in Several Types of Research

In the analysis of the level of awareness of ethical issues in different types of research, nurses had a low level of awareness, while researchers had a high level of awareness. HCPs play an immense role in qualitative research and need skills to observe the participants and interpret the information. In the case of nurses, the most common form of ethical issue in qualitative research arises when the nurse–patient relationship requires therapeutic communication [33]. The participants were highly unaware of the ethical issues involved in conducting research on vulnerable populations. Pregnant women, children, people with mental disabilities, and people from low socio-economic backgrounds are usually considered vulnerable and need extra care when involved in research. Researchers, when carrying out research on vulnerable populations, need to make sure that the participants do not feel pressured and be reassured that participation is voluntary and that they are kept informed about their rights [34]. Also, the results showed that nurses had the lowest level of awareness about the ethical issues involved in various types of research, which is consistent with the survey finding among Japanese nurses that less than 50% of the nurses have knowledge about clinical research design, ethical guidelines, clinical trial regulations, and the vulnerable population [1]. It is inevitable that nurses will come into contact with a patient involved in clinical trial research. Therefore, it is essential for nurses to have a thorough understanding of different types of research and the benefits and risks associated with the research, as nurses in our survey also exhibited the need for more training, which may enhance their understanding of the procedures and ensure patients’ safety [22]. 

### 4.5. Knowledge of Informed Consent and Its Usage

Our next segment of research focused on the level of awareness about informed consent, which is an inevitable part of research ethics. The important aspect of informed consent is to completely inform the participants about the nature of the research, the role of the participant, the objective of the research, the usage of the results, and information about financing bodies [35]. Our results revealed that all the participants had a positive attitude about the importance of informed consent. Physicians had a lower positive attitude about child assent being mandatory in pediatric research (62.8%). This is in line with the survey by Alahmad et al. (2022) [34], where about 71% of the physicians considered obtaining a short assent form that only contains the key information from the children involved in research, and only 6.5% of the physicians considered obtaining children’s assent in a detailed form. Both physicians and researchers had a lower positive attitude about the return of research results to participants (62.5 and 40.4%, respectively). This is consistent with the study by McElfish et al. (2018) [36], which states that concerns about the potential negative emotional impact on participants, participants’ difficulty understanding results, and the consumption of resources, including money and clinician time to complete dissemination were the three most frequently reported challenges to sharing results, whereas the study reported by Wilkins et al. (2019) [37] states that the majority of research participants indicated that receiving research results would be valuable (78.5%) and would increase their likelihood of participating in research (72.4%) and trusting researchers (70.3%). 

### 4.6. Knowledge of the Existence and Significance of IRB Statements

Finally, we evaluated the level of awareness regarding the significance of the IRB. The IRB provides protection for human research participants and performs stringent supervision on the methods followed by research involving human subjects [38]. The statement that “every research project should be evaluated by the full board” had a low positive rating among researchers (35.42%). This may have been considered a cumbersome process. In a survey by Rajab et al. (2019) [39], participants reported that the review process is tedious and time-consuming and the guidelines are ambiguous. On average, the IRBs review 40 protocols of clinical trials annually, and the duration of each meeting is around 45–120 min. The average number of days taken for the full committee review is 20.5 days [40]. This might be the reason that researchers are reluctant to have the full board review every project. Also, researchers had the least positive attitude toward the statement that “the IRB should have an unaffiliated member (non-medical person)” (47.92%). In a survey by Al Fattan et al. (2022) [40], it was reported that only 30.7% of the members of the IRB in Saudi Arabia were non-scientific members. In a survey conducted among non-affiliated IRB members, 88% agreed that having nonscientist IRB members is one of the reasons for making research more accountable to the public [41]. In a study conducted in Bolivia among health researchers, 16% of respondents admitted that they did not follow ethical standards when conducting their research, and 66% of respondents said their institutions did not routinely demand ethics approval for research, leading to the misconduct of research [42]. In a review of Saudi medical journals published between 1979 and 2007 regarding the documentation of ethical conduct, such as obtaining IRB approval and consent and following ethical guidelines, only 0.9% of the studies documented that the ethical guidelines were followed. Also, only 8.6% (*n* = 821) of the studies documented ethics approval from the IRB and obtained informed consent. These rates were seen increasing in the articles published after the year 2000. This suggests that the editors’ lack of rigor and/or investigators’ ignorance of guidelines have been reduced, and the importance of guidelines is being implemented and improved [43].

Our results reveal that there was a significant correlation between profession and awareness of research ethics. Comparatively, researchers had a high level of knowledge regarding research ethics and ethical issues yet a low positive attitude toward key principles of research ethics. This may also be a result of a higher percentage of researchers having undergone research training. A higher level of awareness was seen among all the participants regarding the statements about informed consent and IRB. Though nurses have a positive attitude toward the principles of human research ethics and informed consent, their level of knowledge regarding guidelines and ethical issues is inadequate. This may be due to the fact that a very low percentage of nurses have received education on research ethics. In a survey by Ahn et al. (2020) [44], only 54% of the participants (nurses) agreed that at least one research ethics training course was held annually. In a qualitative analysis reported by Lee et al. (2020) [45], nursing students stated that there was a lack of understanding of how to apply the ethical theories and guidelines they had learned in the classroom to clinical settings. Students noticed a discrepancy between theory and practice and had trouble identifying and judging ethical difficulties in clinical practice. We conclude that there exists a deficit of ethics education in the nursing curriculum, and just like doctors and researchers, nurses also play an equally important role in research in clinical settings. As a result, it is critical to raise their level of awareness of research ethics in order to avoid misconduct.

Some limitations were identified in our analysis. Due to the limited sample size and lack of ethnic diversity, the generalizability of our findings may be limited. To have a comprehensive understanding of the awareness, knowledge, and attitude toward research ethics, it is imperative to conduct future studies utilizing larger sample sizes and including other ethnic groups. Furthermore, an evaluation of the present number of professionals engaged in research and their affiliation with an ethics-trained researcher within a team was not conducted. 

## 5. Conclusions

This study explored the knowledge, awareness, and attitude toward various dimensions of research and research ethics, which include national and international regulatory texts about the protection of human research participants, key principles, types of research, informed consent, and IRB. Researchers were well-versed in research ethics, regulatory guidelines, and ethical issues in various types of research, although they had a very low positive attitude toward the key principles guiding the protection of human research subjects and toward informed consent, whereas nurses were enthusiastic about key principles in human research and the importance of informed consent. Almost all the participants had a positive attitude about the importance of the IRB. Nurses had a low level of awareness regarding the important regulatory guidelines and issues in various types of research and expressed the need for more training in research ethics. Because nurses’ involvement in research is unavoidable, it is equally important to increase their understanding of research ethics. A very low percentage of nurses had received research ethics education. As a result, we emphasize the critical importance of including research ethics education in nursing curricula. Also, the level of knowledge and awareness among physicians is considerably low. As a result, it is critical to hold regular workshops and provide training using real-world case studies to deepen physicians’ understanding of research ethics. Also, periodic workshops should enhance the practical knowledge of professionals regarding the guidelines and guide them in overcoming the practical difficulties they encounter during their practice.

## Figures and Tables

**Table 1:** Demographic characteristics of participants.

	Nurses *n* = 114	Physician *n* = 89	Researchers *n* = 48	Overall *n* = 251
Age mean ± S.D.	34.50 ± 7.66	32.76 ± 10.09	36.80 ± 9.68	34.25 ± 9.07
Gender *n* (%)				
Female	104 (91.23)	36 (40.91)	22 (45.83)	162 (64.54)
Male	10 (8.77)	52 (59.09)	26 (54.17)	88 (35.05)
Marital Status *n* (%)				
Single	47 (41.23)	38 (43.68)	21 (44.68)	106 (42.23)
Married	65 (57.02)	45 (51.72)	24 (51.06)	134 (53.39)
Divorced	2 (1.75)	4 (4.60)	2 (4.26)	8 (3.19)
Widow\er	0	0	0	0
Having children *n* (%)				
Yes	48 (42.11)	39 (45.35)	24 (51.06)	111 (44.22)
Educational level *n* (%)				
Bachelor	102 (91.89)	55 (63.22)	11 (22.92)	168 (66.93)
Master	9 (8.11)	15 (17.24)	10 (20.83)	34 (13.55)
PhD	0	17 (19.54)	27 (56.25)	44 (17.53)
Specialty				
Clinical	12 (10.62)	69 (83.13)	10 (22.22)	91 (36.25)
Basic Science	0	14 (16.87)	35 (77.78)	49 (19.52)
Nursing	101 (89.38)	0	0	101 (40.24)
Training in research ethics *n* (%)				
Yes	36 (31.86)	61 (69.32)	38 (79.17)	135 (53.78)
Training type *n* (%)				
Internet-based	2 (5.41)	9 (14.29)	2 (5.26)	13 (9.62)
Workshop	7 (18.92)	9 (14.29)	2 (5.26)	18 (13.33)
During internship	6 (16.22)	4 (6.35)	2 (5.26)	12 (8.89)
In academic milieu	11 (29.73)	26 (41.27)	8 (21.05)	45 (33.33)
Others	3 (8.11)	2 (3.17)	1 (2.63)	6 (4.44)
More than one type	8 (21.62)	13 (20.63)	23 (60.53)	44 (32.59)
Satisfaction with training *n* (%)				
Very satisfied	13 (36.11)	20 (32.26)	20 (52.63)	53 (39.25)
Moderately satisfied	16 (44.44)	32 (51.61)	16 (42.11)	64 (47.41)
Less satisfied	6 (16.67)	7 (11.29)	1 (2.63)	14 (10.37)
Not satisfied	1 (2.78)	3 (4.84)	1 (2.63)	5 (3.70)
More training needed *n* (%)				
Yes	29 (80.56)	47 (77.05)	30 (78.95)	106 (78.51)
Want to receive training *n* (%)				
Yes	44 (73.33)	29 (85.29)	11 (78.57)	84 (72.41)
Research ethics in your program *n* (%)				
Yes	28 (26.42)	32 (37.21)	26 (54.17)	86 (34.26)
Topics taught *n* (%)				
Research Ethics	8 (53.33)	16 (69.57)	7 (46.67)	31 (36)
Non-research Ethics	6 (40.00)	4 (17.39)	3 (20.00)	13 (15.11)
Both	1 (6.67)	3 (13.04)	5 (33.33)	9 (10.46)
PI or Co-PI *n* (%)				
Yes	7 (6.42)	45 (52.33)	28 (58.33)	80 (31.87)

**Table 2:** Awareness of respondents on the existence and content of key international and local regulatory texts guiding the protection of human research participants.

	Nurses			Physicians			Researchers			*p*-Value
	Not Aware *n* (%)	Simple Aware *n* (%)	Fully Aware *n* (%)	Not Aware *n* (%)	Simple Aware *n* (%)	Fully Aware *n* (%)	Not Aware *n* (%)	Simple Aware *n* (%)	Fully Aware *n* (%)	
Nuremberg Code	96 (84.21)	10 (8.77)	8 (7.02)	55 (64.71)	18 (21.18)	12 (14.12)	24 (50.00)	13 (27.08)	11 (22.92)	0.0002 ^†^*
Declaration of Helsinki	87 (76.32)	16 (14.04)	11 (9.65)	46 (53.49)	23 (26.74)	17 (19.77)	11 (22.92)	19 (39.58)	18 (37.50)	<0.0001 ^†^*
International Ethical Guidelines for Biomedical Research	66 (58.41)	32 (28.32)	15 (13.27)	27 (31.76)	36 (42.35)	22 (25.88)	5 (10.42)	16 (33.33)	27 (56.25)	<0.0001 ^†^*
Belmont Report	98 (88.29)	8 (7.21)	5 (4.50)	62 (73.81)	16 (19.05)	6 (7.14)	26 (55.32)	10 (21.28)	11 (23.40)	<0.0001 ^†^*
Good clinical practice (GCP)	91 (79.82)	15 (13.16)	8 (7.02)	47 (54.65)	23 (26.74)	16 (18.60)	12 (25.53)	22 (46.81)	13 (27.66)	<0.0001 ^†^*
Regulations of the Law of Ethics of Research on Living Creatures in Saudi Arabia	90 (78.95)	16 (14.04)	8 (7.02)	46 (54.12)	23 (27.06)	16 (18.82)	18 (37.50)	18 (37.50)	12 (25.00)	<0.0001 ^†^*
Saudi FDA Regulation	48 (42.86)	35 (31.25)	29 (25.89)	21 (24.42)	33 (38.37)	32 (37.21)	3 (6.25)	26 (54.17)	19 (39.58)	<0.0001 ^†^*
International Ethical Guidelines for Biomedical Research Involving Human Subjects-Islamic view	80 (70.80)	21 (18.58)	12 (10.62)	41 (48.24)	24 (28.24)	20 (23.53)	16 (33.33)	15 (31.25)	17 (35.42)	<0.0001 ^†^*

^†^ Chi-square; * *p*-value is significant at <0.05.

**Table 3:** Respondents’ attitude toward the existence, significance, and capacity to implement key principles guiding the protection of human research subjects (very important and important).

	Nurses *n* (%)	Physicians *n* (%)	Researchers *n* (%)	*p*-Value
Respecting human dignity	111 (44.58)	87 (34.94)	45 (18.07)	0.1399 ^‡^
Benefit	108 (43.55)	85 (34.27)	42 (16.94)	0.0759 ^‡^
Do no harm	109(43.78)	86 (34.54)	44 (17.67)	0.2763 ^‡^
Respect of autonomy	109 (44.31)	83 (33.74)	42 (17.07)	0.0342 ^‡^*
Respect for human vulnerability	108 (43.37)	85 (34.14)	46 (18.47)	0.9183 ^‡^
Respect privacy and confidentiality	109 (43.78)	86 (34.54)	45 (18.07)	0.5001 ^‡^
Respect justice	111 (44.58)	85 (34.14)	45 (18.07)	0.1074 ^‡^
Prevent discrimination	108 (43.55)	82 (33.06)	44 (17.74)	0.3406 ^‡^
Prevent stigmatization	108 (43.72)	83 (33.60)	42 (17.00)	0.2201 ^‡^
Respect for cultural diversity and pluralism	109 (44.13)	81 (32.79)	43 (17.41)	0.1018 ^‡^
Respect for solidarity and cooperation	109 (43.95)	80 (32.26)	42 (16.94)	0.0337 ^‡^*
Respect for social responsibility and health	107 (42.97)	83 (33.33)	45 (18.07)	0.7661 ^‡^
Respect for sharing of benefits	109 (43)	79 (33)	43 (17)	
Protecting future generations	108 (43.37)	82 (32.93)	45 (18.07)	0.3924 ^‡^
Protection of the environment, the biosphere and biodiversity	108 (43.55)	82 (33.06)	44 (17.74)	0.3406 ^‡^

^‡^ Fisher’s Exact Test; * *p*-value is significant at <0.05.

**Table 4:** Awareness among respondents of the existence of ethical issues in several types of research.

	Nurses			Physicians			Researchers			*p*-Value
	Not Aware *n* (%)	Simple Aware *n* (%)	Fully Aware *n* (%)	Not Aware *n* (%)	Simple Aware *n* (%)	Fully Aware *n* (%)	Not Aware *n* (%)	Simple Aware *n* (%)	Fully Aware *n* (%)	
Ethical issues in quantitative research	43 (37.72)	43 (37.72)	28 (24.56)	22 (25.00)	39 (44.32)	27 (30.68)	5 (10.42)	20 (41.67)	23 (47.92)	0.0026 ^‡^*
Ethical issues in qualitative research	41 (36.28)	41 (36.28)	31 (27.43)	22 (25.00)	40 (45.45)	26 (29.55)	7 (14.58)	17 (35.42)	24 (50.00)	0.0132 ^‡^*
Ethical issues in retrospective research	50 (44.25)	44 (38.94)	19 (16.81)	22 (25.00)	32 (36.36)	34 (38.64)	8 (16.67)	18 (37.50)	22 (45.83)	<0.0001 ^‡^*
Ethical issues in clinical trials and interventional studies	44 (39.29)	46 (41.07)	22 (19.64)	17 (19.32)	37 (42.05)	34 (38.64)	4 (8.33)	18 (37.50)	26 (54.17)	<0.0001 ^‡^*
Ethical issues in genetic research	53 (46.90)	42 (37.17)	18 (15.93)	23 (26.44)	37 (42.53)	27 (31.03)	4 (8.33)	20 (41.67)	24 (50.00)	<0.0001 ^‡^*
Ethical issues in vulnerable populations	55 (48.67)	38 (33.63)	20 (17.70)	25 (28.41)	34 (38.64)	29 (32.95)	9 (18.75)	19 (39.58)	20 (41.67)	0.0006 ^‡^*

^‡^ Fisher’s Exact Test; * *p*-value is significant at <0.05.

**Table 5:** Attitudes of physicians, nurses, and researchers toward informed consent and its usage.

	Nurses			Physicians			Researchers			*p*-Value
	Always *n* (%)	Sometimes *n* (%)	Never *n* (%)	Always *n* (%)	Sometimes *n* (%)	Never *n* (%)	Always *n* (%)	Sometimes *n* (%)	Never *n* (%)	
Informed consent is mandatory	100 (88.50)	11 (9.73)	2 (1.77)	64 (72.73)	24 (27.27)	0 (0.00)	34 (70.83)	11 (22.92)	3 (6.25)	0.0010 ^‡^*
Child assent is mandatory in pediatric research	84 (74.34)	25 (22.12)	4 (3.54)	54 (62.79)	28 (32.56)	4 (4.65)	31 (65.96)	10 (21.28)	6 (12.77)	0.0889 ^‡^
The informed consent should include a section stating the purpose of the study	100 (88.50)	10 (8.85)	3 (2.65)	75 (85.23)	10 (11.36)	3 (3.41)	36 (75.00)	5 (10.42)	7 (14.58)	0.0540 ^‡^*
A statement that participation is voluntary	99 (87.61)	11 (9.73)	3 (2.65)	67 (77.01)	13 (14.94)	7 (8.05)	35 (72.92)	6 (12.50)	7 (14.58)	0.0446 ^‡^*
A statement of respect for privacy and confidentiality	102 (90.27)	7 (6.19)	4 (3.54)	78 (88.64)	7 (7.95)	3 (3.41)	38 (80.85)	2 (4.26)	7 (14.89)	0.0853 ^‡^
A description of any reasonably foreseeable risks or discomforts to the subject	98 (86.73)	11 (9.73)	4 (3.54)	75 (85.23)	10 (11.36)	3 (3.41)	38 (79.17)	3 (6.25)	7 (14.58)	0.0942 ^‡^
A description of any benefits to the subject or to others	96 (84.96)	10 (8.85)	7 (6.19)	62 (70.45)	22 (25.00)	4 (4.55)	24 (50.00)	17 (35.42)	7 (14.58)	<0.0001 ^‡^*
A disclosure of appropriate alternative procedures or courses of treatment	97 (85.84)	10 (8.85)	6 (5.31)	67 (76.14)	17 (19.32)	4 (4.55)	27 (56.25)	12 (25.00)	9 (18.75)	0.0006 ^‡^*
An explanation of compensation	90 (79.65)	14 (12.39)	9 (7.96)	58 (65.17)	26 (29.21)	5 (5.62)	25 (52.08)	18 (37.50)	5 (10.42)	0.0026 ^†^*
An explanation of whom to contact for answers in the event of a research-related injury	98 (86.73)	9 (7.96)	6 (5.31)	78 (87.64)	8 (8.99)	3 (3.37)	35 (72.92)	8 (16.67)	5 (10.42)	0.1766 ^‡^
A statement about unexpected risks	97 (85.84)	11 (9.73)	5 (4.42)	66 (74.16)	15 (16.85)	8 (8.99)	38 (79.17)	3 (6.25)	7 (14.58)	0.0608 ^†^
An explanation of estimated circumstances under which the subject’s participation may be terminated	94 (82.46)	13 (11.40)	7 (6.14)	72 (80.90)	14 (15.73)	3 (3.37)	29 (60.42)	15 (31.25)	4 (8.33)	0.0204 ^‡^*
An explanation about any additional costs to the subject that may result from participation in the research	93 (81.58)	15 (13.16)	6 (5.26)	62 (69.66)	19 (21.35)	8 (8.99)	27 (56.25)	11 (22.92)	10 (20.83)	0.0060 ^†^*
An explanation about the consequences of a subject’s decision to withdraw	93 (81.58)	14 (12.28)	7 (6.14)	68 (76.40)	14 (15.73)	7 (7.87)	27 (56.25)	12 (25.00)	9 (18.75)	0.0141 ^†^*
A statement about the return of research results to participants	92 (80.70)	17 (14.91)	5 (4.39)	55 (62.50)	25 (28.41)	8 (9.09)	19 (40.43)	21 (44.68)	7 (14.89)	<0.0001 ^†^*

^†^ Chi-square; ^‡^ Fisher’s Exact Test; * *p*-value is significant at <0.05.

**Table 6:** Respondents’ attitude toward the existence and significance of the IRB statements (strongly agree and agree).

	Nurses *n* (%)	Physicians *n* (%)	Researchers *n* (%)	Total *n* (%)	*p*-Value
Every research should be evaluated by the IRB	87 (76.32)	78 (88.64)	31 (64.58)	196 (78.57)	<0.0001 ^†^*
Every research should be evaluated by the full board	87 (76.32)	54 (62.07)	17 (35.42)	158 (63.35)	<0.0001 ^†^*
The IRB chairman can approve research proposals without referring them to the full board	53 (46.49)	38 (43.18)	23 (47.92)	114 (45.24)	0.0186 ^†^*
The IRB can approve, disapprove, or ask for modifications	84 (73.68)	75 (85.23)	38 (79.17)	197 (78.97)	0.1403 ^‡^
Five members be available during each meeting	79 (69.30)	34 (39.08)	28 (58.33)	141 (56)	0.0004 ^†^*
One member has knowledge of research methods	68 (59.65)	64 (73.56)	35 (72.92)	167 (66.93)	0.0803 ^†^
One member has knowledge of bioethics	65 (74.71)	65 (74.71)	37 (77.08)	174 (69.72)	0.2390 ^†^
One member be specialized in the medical field	79 (69.91)	69 (80.23)	32 (66.67)	180 (72.69)	0.0474 ^†^*
The IRB should have an unaffiliated member (non-medical person)	73 (64.04)	48 (55.17)	23 (47.92)	144 (57.77)	0.3464 ^†^
One member should be aware of the local culture	85 (74.56)	71 (81.61)	35 (72.92)	191 (76.89)	0.5203 ^†^
The IRB should have two genders	79 (69.91)	39 (44.83)	24 (50.00)	142 (57.2)	0.0014 ^†^*
The IRB should avoid any conflict of interest	88 (79.28)	75 (86.21)	40 (83.33)	203 (82.25)	0.0005 ^‡^*
It is the responsibility of the IRB to review and evaluate the scientific design and conduct of the research	87 (76.99)	66 (75.86)	28 (58.33)	181 (73.2)	0.0079 ^†^*
It is the IRB responsibility to review and evaluate the confidentiality issues in research	89 (78.76)	73 (83.91)	38 (79.17)	200 (80.8)	0.5268 ^†^
It is the IRB responsibility to review and evaluate the contents of the informed consent	87 (77.68)	73 (83.91)	41 (85.42)	201 (81.52)	0.4500 ^‡^
It is the IRB responsibility to review and evaluate the process of collecting informed consent	88 (78.57)	67 (77.01)	38 (79.17)	193 (78.32)	0.0412 ^†^*
It is the IRB responsibility to take into account the community considerations	86 (76.11)	67 (77.01)	34 (70.83)	187 (75.6)	0.7977 ^†^
It is the IRB responsibility to review and evaluate the care and protection of research participants	88 (77.88)	73 (83.91)	40 (83.33)	201 (80.8)	0.8522 ^‡^
It is the IRB responsibility to review and evaluate the recruitment of research participants	88 (77.88)	65 (75.58)	33 (68.75)	186 (75.1)	0.7158 ^†^

^†^ Chi-square; ^‡^ Fisher’s Exact Test; * *p*-value is significant at <0.05.

## Data Availability

Parts of the datasets generated during the study are available from the corresponding author upon reasonable request.
